# Effects of corticosteroids on severe community-acquired pneumonia: a closer look at the evidence

**DOI:** 10.1186/s13054-023-04614-3

**Published:** 2023-08-29

**Authors:** Cho-Han Chiang, Xin Ya See, Tsu Hsien Wang, Yu-Cheng Chang, Jui-En Lo, Wei-Tao Liu, Cheryn Yu Wei Choo, Cho-Hsien Chiang, Yuan Ping Hsia, Cho-Hung Chiang

**Affiliations:** 1 Department of Medicine, Mount Auburn Hospital, Harvard Medical School, Cambridge, MA USA; 2 Department of Medicine, Unity Hospital, Rochester Regional Health, Rochester, NY USA; 3 Department of Emergency Medicine, Taipei Tzu Chi Hospital, Buddhist Tzu Chi Foundation, New Taipei City, Taiwan; 4 Department of Medicine, Danbury Hospital, Danbury, CT USA; 5 School of Medicine, National Taiwan University College of Medicine, Taipei, Taiwan; 6 Department of Internal Medicine, Singapore Health Services, Singapore, Singapore; 7 Department of Environmental and Occupational Medicine, National Taiwan University Hospital, Taipei, Taiwan; 8 London School of Hygiene and Tropical Medicine, London, UK; 9 Department of Family Medicine, Taipei Tzu Chi Hospital, Buddhist Tzu Chi Foundation, No. 289, Jianguo Rd., Xindian Dist., New Taipei City, 231405 Taiwan; 10 Department of Internal Medicine, National Taiwan University Hospital, Taipei, Taiwan

## To the Editor,

We read with interest the article published by Wu et al., who reported that adjunctive corticosteroids can provide survival benefits and improve clinical outcomes without increasing adverse events in patients with severe community-acquired pneumonia (sCAP) [1]. We commend the authors for conducting this comprehensive systematic review and meta-analysis on this crucial topic, as previous randomized controlled trials have yielded conflicting results. However, we have several concerns regarding the methodologies and results presented in this paper. 

**Fig. 1:**
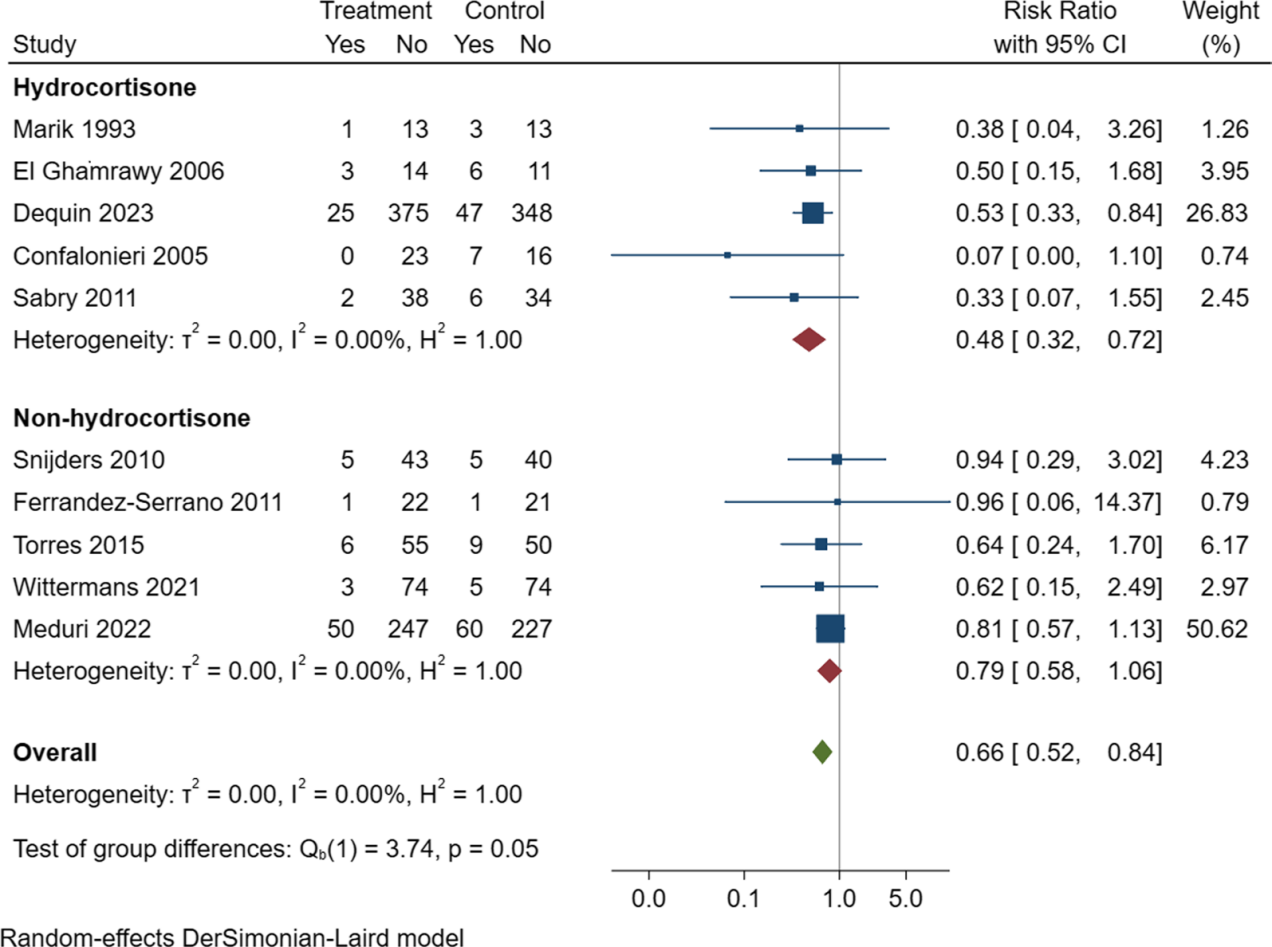
Forest plot summarizing the effects of different types of corticosteroids on all-cause mortality

First, this meta-analysis did not include three pivotal trials: the Santeon-CAP Trial [2], the CAPISCE-Trial [3], and the Bellvitge Trial [4]. While these trials recruited a mixed population of patients with severe and non-severe CAP, all of them reported subgroup analyses of patients with sCAP, which could be utilized for data extraction in a study-level meta-analysis. In each of their respective subgroup analyses, the Santeon-CAP Trial, CAPISCE-Trial, and the Bellvitge Trial did not find mortality benefits associated with dexamethasone, prednisolone, or methylprednisolone, respectively, among patients hospitalized for sCAP. We extracted the data from these three trials and conducted a meta-analysis by pooling the results from all 10 studies (7 studies from the meta-analysis by Wu et al. and 3 studies identified through our literature search). We found that hydrocortisone was associated with a reduction in all-cause mortality (HR 0.48 [95% CI: 0.32–0.72]), but this observation was not seen for non-hydrocortisone corticosteroids (HR 0.79 [95% CI: 0.58–1.06]) (Fig. 1). Based on these results, it appears that only hydrocortisone, but not other corticosteroids, exhibited an association with a reduced mortality risk among patients hospitalized for sCAP.

Second, the authors reported that patients who received corticosteroids, particularly hydrocortisone, for a duration of ≤ eight days without tapering, experienced significantly lower mortality risks (as demonstrated in Table 3 of the manuscript). However, the CAPE COD trial conducted by Dequin and colleagues, which reported mortality benefits among patients receiving hydrocortisone, used an initial hydrocortisone dose of 200 mg with a gradual taper over 8 or 14 days [5]. This study appeared to have been inaccurately categorized under the subgroup analysis of corticosteroids administered for a duration of ≤ eight days without tapering. We performed a subgroup analysis by re-classifying the CAPE COD trial [5] under the subgroup of duration of > eight days with tapering and included the three newly identified studies (2–4). We found that corticosteroid durations of over 8 days or with a taper showed a similar reduction in all-cause mortality compared to corticosteroid durations of less than 8 days or without a taper (HR 0.69 [95% CI: 0.51–0.93] vs. HR 0.55 [95% CI: 0.33–0.92]). Thus, in contrast to the authors' findings, the duration or tapering of corticosteroids did not appear to affect mortality benefits.

Third, the authors did not report on the risk of hyperglycemia, a significant adverse event associated with corticosteroid use. We conducted an updated meta-analysis that encompassed all studies reporting on hyperglycemia and found an approximately 50% increased risk of hyperglycemia associated with corticosteroid use compared to placebo (HR 1.50 [95% CI: 1.04–2.17]). These findings further substantiate that corticosteroids can elevate the risk of hyperglycemia, necessitating caution, particularly in diabetes patients hospitalized for sCAP.

Finally, the authors omitted the assessment of certainty or confidence in the body of evidence for each evaluated outcome. This represents a crucial element of the Preferred Reporting Items for Systematic Reviews and Meta-analyses (PRISMA) Checklist, which is recommended for meta-analyses of randomized controlled trials. Such an evaluation contextualizes the results of a meta-analysis and facilitates their application to clinical practice.

Once again, we commend the authors for undertaking this significant work and hope that our comments contribute additional insights to the presented data.

## Data Availability

The authors confirm that the data supporting the findings of this study are available within the article. The data used for all analyses including the analytic code can be obtained upon reasonable request from the corresponding author.
